# A new zebrafish model produced by TILLING of SOD1-related amyotrophic lateral sclerosis replicates key features of the disease and represents a tool for *in vivo* therapeutic screening

**DOI:** 10.1242/dmm.012013

**Published:** 2013-10-02

**Authors:** Marc M. J. Da Costa, Claire E. Allen, Adrian Higginbottom, Tennore Ramesh, Pamela J. Shaw, Christopher J. McDermott

**Affiliations:** Sheffield Institute for Translational Neuroscience, The University of Sheffield, Sheffield S10 2HQ, UK.

**Keywords:** MND, ALS, SOD1, Zebrafish

## Abstract

Mutations in the superoxide dismutase gene (*SOD1*) are one cause of familial amyotrophic lateral sclerosis [ALS; also known as motor neuron disease (MND)] in humans. ALS is a relentlessly progressive neurodegenerative disease and, to date, there are no neuroprotective therapies with significant impact on the disease course. Current transgenic murine models of the disease, which overexpress mutant SOD1, have so far been ineffective in the identification of new therapies beneficial in the human disease. Because the human and the zebrafish (*Danio rerio*) SOD1 protein share 76% identity, TILLING (‘targeting induced local lesions in genomes’) was carried out in collaboration with the Sanger Institute in order to identify mutations in the zebrafish *sod1* gene. A T70I mutant zebrafish line was characterised using oxidative stress assays, neuromuscular junction (NMJ) analysis and motor function studies. The T70I *sod1* zebrafish model offers the advantage over current murine models of expressing the mutant Sod1 protein at a physiological level, as occurs in humans with ALS. The T70I *sod1* zebrafish demonstrates key features of ALS: an early NMJ phenotype, susceptibility to oxidative stress and an adult-onset motor neuron disease phenotype. We have demonstrated that the susceptibility of T70I *sod1* embryos to oxidative stress can be used in a drug screening assay, to identify compounds that merit further investigation as potential therapies for ALS.

## INTRODUCTION

Amyotrophic lateral sclerosis (ALS) is a devastating disease that is currently incurable. Typical features include progressive muscle weakness and wasting, caused by the death of motor neurons in the motor cortex, brainstem and spinal cord. The majority of patients survive between 2 and 5 years following the onset of symptoms (Ferraiuolo et al., 2011). The current approach to drug identification for ALS has not delivered an effective intervention. Riluzole is the only disease-modifying therapy available and its effect is modest, with survival extension of only ~3 months (Bensimon et al., 1994).

Although the majority of ALS cases are sporadic, 5–10% are familial (fALS). Mutations in the superoxide dismutase 1 gene (*SOD1*) account for 20% of fALS cases; until the recent discovery of a large hexanucleotide repeat expansion in the first intron of C9ORF72 (DeJesus-Hernandez et al., 2011; Renton et al., 2011), such mutations were thought to be the leading cause of fALS. Mutations in *SOD1* have been implicated in ALS since 1993, and mutant *SOD1* mice and *in vitro* models have been the dominant models employed to investigate pathogenesis and potential therapies for ALS (Rosen et al., 1993). Several pathogenic mechanisms have been proposed for ALS that is caused by SOD1 mutation, including glutamate excitotoxicity, oxidative stress, mitochondrial dysfunction and axonal defects, including the loss of the neuromuscular junction (NMJ) (Ferraiuolo et al., 2011). Oxidative stress is a mechanism of particular interest owing to the normal role that SOD1 plays in the cell as a cytoplasmic free-radical scavenger (Barber and Shaw, 2010). Current evidence suggests that mutation in SOD1 confers a toxic gain of function in ALS (Dal Canto and Gurney, 1994), rather than a loss of function (Reaume et al., 1996), and a component of this toxicity disrupts the normal handling of free radicals by the cell, generating oxidative stress.

Current *in vivo* models of ALS rely on the overexpression of mutant SOD1. The most widely used transgenic mutant *SOD1* mouse model develops a very early and aggressive phenotype and, although the mutant mice develop progressive hind limb weakness leading to paralysis and death, with very predictable disease progression (Tu et al., 1996), the accelerated time course of the disease does not accurately reflect the human disease. Furthermore, many of the therapies that have seemed to be neuroprotective in the overexpressing transgenic mouse model have failed to translate into beneficial effects in human trials (Benatar, 2007; Gordon et al., 2007). There are several potential reasons for this poor translation of benefit into the human disease. Many of the murine trials have been under-powered and with inadequate attention to the potential confounding effects of gender, litter, heterogeneity of genetic background and pre-symptomatic treatment administration. Furthermore, the relevance of mouse SOD1 models to ALS more broadly has been questioned, given the presence of TDP-43-positive inclusions in motor neurons from most cases of ALS, which are absent in the SOD1-related disease subtype (Mackenzie et al., 2007). However, despite these shortcomings, the transgenic *SOD1* mouse model of ALS has been extensively used for preclinical testing (Turner and Talbot, 2008; Knippenberg et al., 2010). New approaches to therapy development are urgently required, and an *in vivo* system allowing rapid and efficient drug screening would be a useful addition to current models.

The zebrafish represents an alternative model for studying human disease. Zebrafish are capable of producing hundreds of transparent embryos per week, which are externally fertilized and hence easily manipulated using genetic and pharmacological approaches. There are many transgenic models available, some with fluorescent reporters for easy identification of specific cell types and protein expression. Embryos and adults can also be used for behavioural studies and motor function tests. The high fecundity and relatively low maintenance costs mean that high-throughput screens of multiple drug targets is a viable option, and is being increasingly used (Kabashi et al., 2011).

**TRANSLATIONAL IMPACT**

**Clinical issue**

Mutation of the superoxide dismutase 1 (*SOD1*) gene is the second most common cause of familial amyotrophic lateral sclerosis (ALS), a form of motor neuron disease. SOD1 encodes a protein that is responsible for reducing free oxygen radicals in the cytoplasm, and expression of mutant SOD1 can lead to oxidative stress, which could form the basis of motor neuron death in ALS. The disease, which has devastating effects on muscle function throughout the body, is currently incurable, and the only drug available has a very modest effect on disease course. Further research into identifying potential therapies for ALS is needed, particularly because efforts based on transgenic mouse models have thus far been ineffective. Zebrafish have huge potential for high-throughput drug screening because of their small size and genetic synteny with humans. This study employs a zebrafish model to screen for potential neuroprotective therapies to treat ALS.

**Results**

In collaboration with the Sanger Institute, the authors applied ENU mutagenesis and targeting induced local lesions in genomes (TILLING) to develop a new zebrafish *sod1* mutant. The novel T70I *sod1* model displays late-onset motor symptoms and motor neuron loss, as seen in individuals with ALS. The authors also report that the mutation has a toxic gain-of-function effect, consistent with previous data on other SOD1 mutations. They also show that homozygous T70I *sod1* mutant embryos have a marked susceptibility to oxidative stress compared with wild-type controls, and demonstrate that this feature can be exploited in a survival assay that could be used for drug screening. Compounds known for their antioxidant properties were tested on the zebrafish embryos in proof-of-principle assays, in which treatment with apomorphine-S provided the biggest increase in survival (66%).

**Implications and future directions**

This work provides a new animal model of ALS and a robust assay with a clear readout that together have the potential for use in high-throughput drug screening. Compounds that have significant effects on survival in the zebrafish could be prioritised for mammalian studies and subsequently in human clinical trials, which could lead to the generation of therapies for the treatment of ALS and related motor neuron diseases. The study also demonstrates the power of TILLING for the rapid development of zebrafish mutants that accurately and reproducibly recapitulate human disease.

There is only one zebrafish orthologue of *SOD1*, and this, combined with the fact that human and zebrafish SOD1 protein share 76% identity, indicates the potential to use zebrafish as an ALS model. The development of motor phenotypes in zebrafish, following transient expression of either mutant SOD1 or mutant TDP-43, further supports this possibility (Lemmens et al., 2007; Kabashi et al., 2008; Kabashi et al., 2010). However, a key advantage of the zebrafish model is the relative ease with which genetic manipulation can be performed, using ethylnitrosourea (ENU) mutagenesis and targeting induced local lesions in genomes (TILLING) (McCallum et al., 2000; Wienholds et al., 2002). These techniques allow the development of a zebrafish model expressing mutant Sod1 protein at normal physiological levels, rather than at the high levels of overexpression seen in most currently available murine transgenic models. Using these techniques we have developed a new zebrafish model of *sod1* ALS that replicates key features of the disease, which allows this model to be used for the screening of potential neuroprotective therapies.

## RESULTS

Through TILLING, the missense mutation T70I was generated in the zebrafish *sod1* gene. The T70I mutation occurs in the zinc-binding loop of the Sod1 protein, next to a highly conserved HGGP motif, which is involved in binding the zinc ion (supplementary material Fig. S1). To confirm that the ENU mutant only carries the *sod1* mutation, the founder was backcrossed with wild-type (WT) AB* zebrafish for six generations, prior to utilizing them for analysis. The *Danio rerio* chromosome 10 is syntenic to some parts of human chromosomes 2, 5, 9, 11, 13 and 21 (Woods et al., 2000; Woods et al., 2005). The human *SOD1* gene is located on chromosome 21, supporting that this synteny is correct. We carefully analysed the *sod1* locus of zebrafish chromosome 10 and observed synteny with human chromosomes 11, 13 and 21. The syntenic map of the zebrafish *sod1* locus to the human chromosome shows that this locus contains no ALS genes that could contribute to the phenotype of these animals. Thus, this allele represents a true *sod1* mutation in the zebrafish *sod1* gene.

### Normal Sod1 expression level, but reduced enzymatic activity, in T70I *sod1* zebrafish

**Fig. 1:**
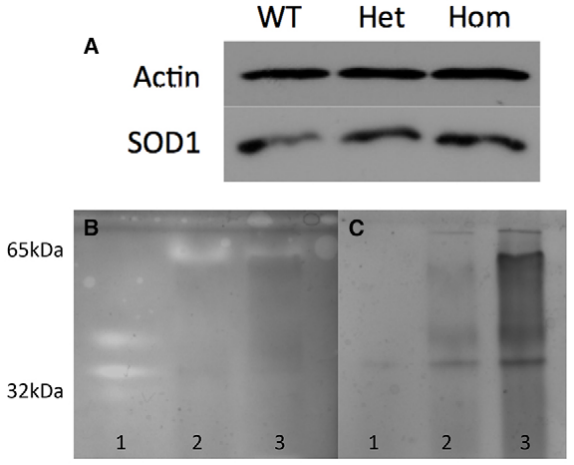
**Normal expression and function of WT and T70I Sod1.** (A) Western blot with Sod1 expression in 72-hpf wild-type (WT), heterozygous T70I (Het) and homozygous T70I (Hom) zebrafish larvae. (B) Native acrylamide gel of NSC34 and 5-dpf zebrafish sonicates. The gel was stained for SOD1 activity using NBT/riboflavin. (C) Native acrylamide gel of NSC34 and 5-dpf zebrafish sonicates. The gel was stained with Coomassie blue to control for whole protein loading. (B,C) Lane 1 contains the sonicates of NSC34 cells expressing WT human SOD1. Lane 2 contains WT *sod1* 5-dpf zebrafish sonicates. Lane 3 contains homozygous T70I *sod1* 5-dpf zebrafish sonicates.

The presence of mutant Sod1, both in heterozygous T70I *sod1* and in homozygous T70I *sod1* embryos at 72 hours post-fertilisation (hpf) does not alter the total expression levels of Sod1 in comparison with WT clutch mates (Fig. 1A). The native gel in Fig. 1B demonstrates that there is SOD1 enzymatic activity in all three of the samples. Despite more protein being loaded into lane 3 (homozygous) than lane 2 (WT), there is an apparent reduction in Sod1 enzymatic activity at 65 kDa and no detectable novel activity elsewhere on the gel (Fig. 1B). This indicates that the T70I mutation causes at least a partial reduction of dismutase function of the Sod1 protein. Western blotting of native gels failed to detect any bands smaller than 65 kDa, despite using two different polyclonal anti-SOD1 antibodies (data not shown), and hence we cannot categorically say that the band at 65 kDa on the native gel with dismutase activity consists solely of Sod1. Instead, the presence of dismutase activity at a higher molecular weight than a monomer indicates that the native gel might include components of Sod1 complexed with itself or with other proteins (Pedrini et al., 2010).

### Zebrafish T70I Sod1 leads to increased motor neuronal oxidative stress *in vitro*

**Fig. 2:**
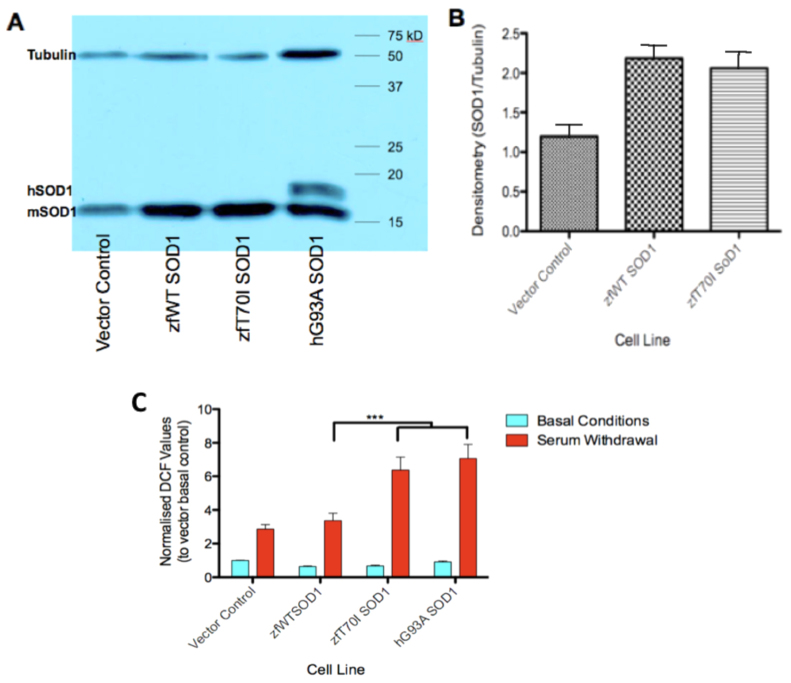
**T70I Sod1 in cell culture.** (A) Western blot with β-tubulin and SOD1 expression in NSC34 cell lines. hSOD1 denotes the level at which human SOD1 runs. mSOD1 denotes the level at which mouse and zebrafish SOD1 run. (B) Densitometric analysis of SOD1 expression in zfWT *sod1* and zfT70I *sod1* NSC34 cell lines compared with the vector control NSC34 cell line. (C) Measure of reactive oxygen species in NSC34 cell lines using DCF fluorescence. The data are means ± s.e.m. (*n*=3); statistical significance of difference between means was determined by two-way ANOVA and Bonferroni post-tests. ***P<0.001. Serum withdrawal was for 6 hours.

Oxidative stress represents one element of the toxicity of mutant SOD1 (Barber and Shaw, 2010). We predicted that expression of zebrafish (zf)T70I Sod1, would lead to similar susceptibility to oxidative stress as observed with the expression of human (h)G93A SOD1, in the NSC34 cellular model (Barber et al., 2006). The molecular weights of zfSod1 and mouse (m)SOD1 proteins are very similar at around 16 kDa, and the bands that they produce on a western blot overlap (Fig. 2A). The zfSod1 protein is present in the NSC34 cell lysates despite the cell culture conditions, with a temperature of 37°C rather than the 28°C temperature normally used for zebrafish maintenance. The densitometric analysis (Fig. 2B) shows that there is almost double the expression of Sod1 in the zfWT *sod1* and zfT70I *sod1* NSC34 cell lines, compared with the vector control NSC34 cell line. This is comparable with expression levels of transfected mutant and hWT SOD1 demonstrated in previous reports (Cookson et al., 2002; Menzies et al., 2002; Barber et al., 2009).

Serum withdrawal proved to be a very robust and consistent method for generating oxidative stress, which differentiated mSod1-expressing NSC34 cells from control cell lines. Upon serum withdrawal for 6 hours, NSC34 cells stably transfected with zfT70I *sod1* or hG93A *SOD1* demonstrated approximately double the oxidative stress levels compared with transfection with zfWT *sod1* or a vector control (*P*<0.001) (Fig. 2C).

### zfT70I Sod1 causes altered NMJ morphology

NMJ staining was carried out on the 11 days post-fertilisation (dpf) progeny resulting from heterozygous T70I mutant *sod1* incrosses. These incrosses gave rise to a normal Mendelian ratio of WT, heterozygous and homozygous zebrafish (1:2:1). No morphological differences were noted between clutch mates.

**Fig. 3:**
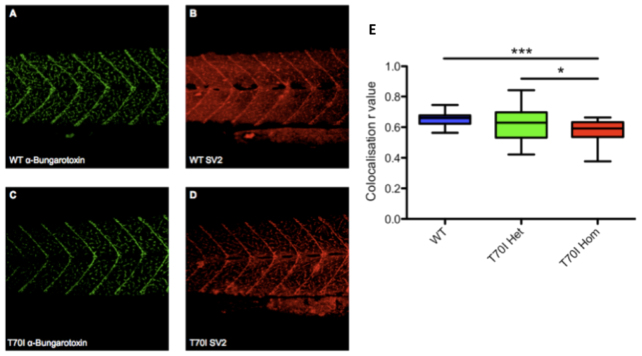
**Neuromuscular junction analysis.** (A) 11-dpf WT larva stained with α-bungarotoxin conjugated with Alexa Fluor 488. (B) 11-dpf WT larva stained with SV2 (from Developmental Studies Hybridoma Bank, University of Iowa, IA, USA) and Alexa Fluor 594. (C) 11-dpf homozygous T70I larva stained with α-bungarotoxin conjugated with Alexa Fluor 488. (D) 11-dpf homozygous T70I larva stained with SV2 and Alexa Fluor 594. Images have been background corrected. (E) Colocalisation analysis was completed using the WCIF suite of plug-ins for ImageJ. Colocalisation *r*-values from the interseptal regions of WT, T70I heterozygous (Het) and T70I homozygous (Hom) 11-dpf larvae from a heterozygous incross. Statistical significance of difference between means was determined by one-way ANOVA and Dunn’s multiple comparison test (*n*=30). *P<0.05; ***P<0.001.

Confocal microscopic analysis of the immunostained zebrafish showed that homozygous T70I zebrafish larvae stained for both α-bungarotoxin (post-synaptic) and SV2 (pre-synaptic) with less intensity than their WT and heterozygous clutch mates (Fig. 3). A significant decrease of ~10% (*P*<0.001) in colocalisation of SV2 and α-bungarotoxin in the interseptal region was observed in 11-dpf homozygous T70I larvae, as compared with their WT clutch mates (Fig. 3E). Heterozygous T70I mutant *sod1* larvae displayed the largest variability in *r*-value (overlap coefficient, where 1 is complete overlap and 0 is no overlap), and there was no significant difference between the *r*-values of WT and heterozygous larvae. The decrease in *r*-value corresponds to a defect of the NMJ.

### zfT70I Sod1 causes a reduction in spinal cord motor neurons in adult zebrafish

Choline acetyltransferase (ChAT) staining is a hallmark feature of cholinergic motor neurons. ChAT immunostaining was performed on the spinal cord sections obtained from 3-year-old zebrafish, resulting from heterozygous T70I mutant *sod1* incrosses. Motor neurons in the zebrafish spinal cord are small or large depending on the maturation stage of the neurons (Becker et al., 2004). A reduction was observed in both the total number of all ChAT-positive motor neurons and a subset of mature ChAT-positive motor neurons in the T70I mutants. Motor neurons were classified as mature if they were over 200 μm^2^.

**Fig. 4:**
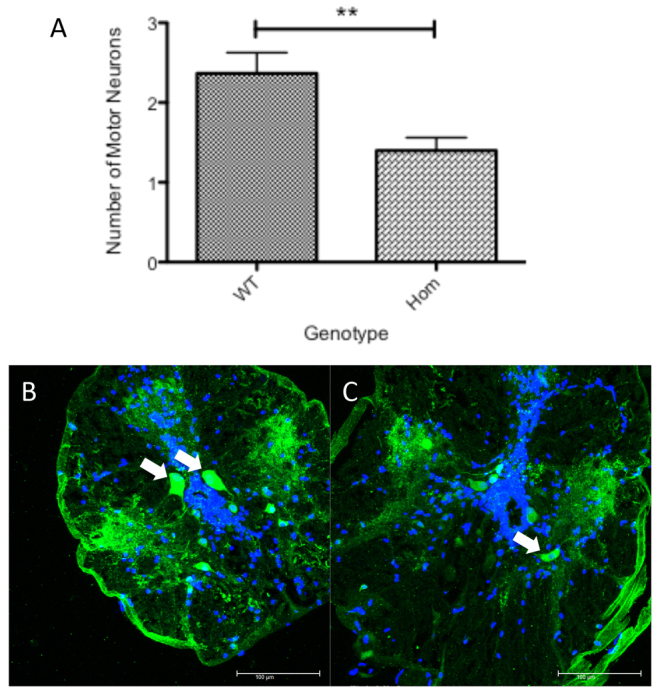
**ChAT staining in adult zebrafish spinal cord.** (A) Bar chart depicting the average number of large motor neurons with a cell body area of >200 μm^2^ per spinal cord section in homozygous T70I *sod1* zebrafish and their WT clutch mates. *n*=120 per group (40 sections per fish; three fish used). Statistical significance was calculated using a two-tailed *t*-test. **P<0.01. (B,C) Confocal images of adult WT zebrafish spinal cord (B) and adult homozygous T70I *sod1* zebrafish spinal cord (C) (ChAT, green; DAPI nuclei, blue). White arrows indicate ChAT-positive large motor neurons (>200 μm^2^). Scale bars: 100 μm.

Confocal microscopic analysis of the immunostained zebrafish spinal cord sections showed a significant reduction (*P*<0.01) by ~50% of large motor neurons with a cell body area of >200 μm^2^ in homozygous T70I zebrafish compared with their WT clutch mates (Fig. 4).

### zfT70I causes motor impairment in adult fish

**Fig. 5:**
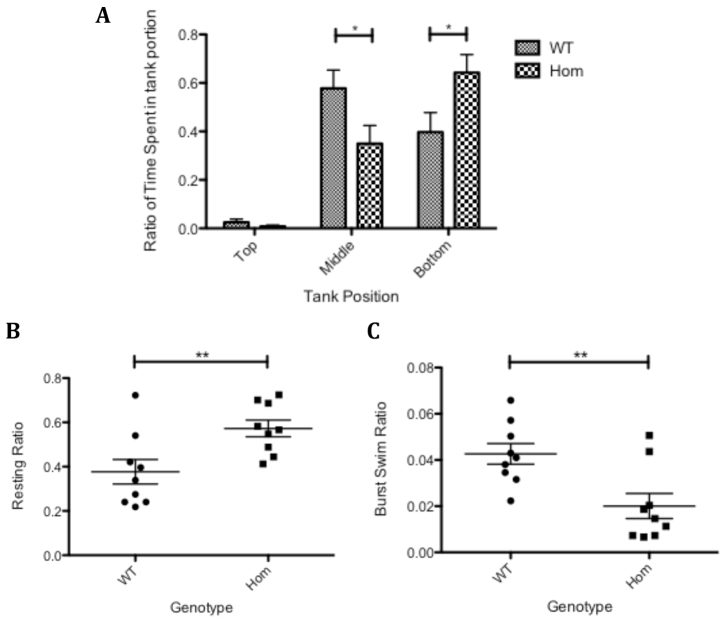
**Movement analysis of T70I Sod1 adult zebrafish.** (A) Bar chart depicting the ratio of time that 20-month-old adult zebrafish spent in a mean positional plane in a fish tank. *n*=10 for each group. Statistical significance calculated using one-way ANOVA and Bonferroni’s multiple comparison test. (B) Graph depicting the ratio of time that 20-month-old adult zebrafish spent in the ‘resting’ phase of swimming activity. Statistical significance was calculated using a two-tailed *t*-test. (C) Graph depicting the ratio of time that 20-month-old adult zebrafish spent in the ‘burst’ phase of swimming activity. Statistical significance was calculated using a two-tailed *t*-test. *P<0.05; **P<0.01.

Motor phenotypes were observed in 20-month-old homozygous T70I *sod1* zebrafish resulting from a heterozygous incross. These motor phenotypes were quantified using the ViewPoint Zebralab system. The ViewPoint Zebralab ‘Tracking System’ readouts demonstrated that the homozygous T70I *sod1* zebrafish spent significantly more time (20%) in the bottom third of the tank, compared with their WT clutch mates (*P*<0.05; Fig. 5A).

The ViewPoint Zebralab ‘Quantitization Program’ was used to quantify the duration of time that the zebrafish spend swimming at fast, moderate and slow speeds. Using this system it was observed that there was a 44% increase in the ratio of time (resting time/total time) (*P*<0.01) that the homozygous T70I *sod1* zebrafish spent in the ‘resting’ phase of swimming activity (Fig. 5B). The ratio of time (burst time/total time) (*P*<0.01) that homozygous T70I *sod1* zebrafish spent in the ‘burst’ phase of swimming activity (Fig. 5C) as compared with their WT clutch mates was decreased by 50%.

### Oxidative stress survival assay as proof of principle for further drug screening

**Fig. 6:**
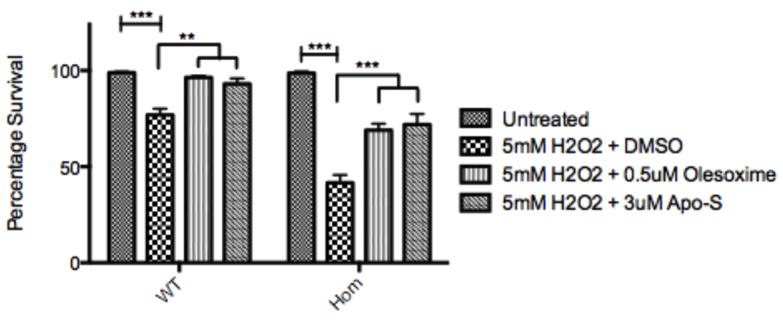
**Bar chart depicting the effects of 5 mM hydrogen peroxide on the survival of WT and T70I homozygous zebrafish embryos with either DMSO, 0.5 μM olesoxime or 3 μM Apo-S.** Data are percentages of survival as judged by the presence of a beating heart. *n*=3. Statistical significance calculated using one-way ANOVA and Bonferroni’s multiple comparison test. **P<0.05; ***P<0.001.

Mutant Sod1 confers significant susceptibility to oxidative stress in embryonic zebrafish, as demonstrated by a large reduction in survival of homozygous T70I *sod1* embryos upon incubation with 5 mM hydrogen peroxide (H_2_O_2_) and 1% DMSO (mean survival 45%, s.d.=15.9) compared with WT embryos (mean survival 80% s.d.=12.3) (Fig. 6).

Compounds known for their antioxidant properties were tested on the zebrafish in proof-of-principle assays in order to determine the suitability of the assay for a more high-throughput drug screen in the future. All of the drugs used in the survival assay were trialled at a range of concentrations to construct dose-response curves, from which the most successful (activity versus toxicity) concentrations were taken forward (0.5 μM to 3 μM).

Olesoxime (optimal concentration 0.5 μM) and apomorphine-S (optimal concentration 3 μM) were demonstrated to protect both the WT (by 22% and 19%, respectively) and the T70I homozygous *sod1* (by 62% and 66%, respectively) embryos from oxidative stress (homozygous recovery *P*<0.001, WT recovery *P*<0.01) (Fig. 6). The robust difference observed between WT and homozygous T70I *sod1* zebrafish embryos in their ability to cope with oxidative stress provides a useful readout for a drug screen of compounds to ameliorate oxidative stress.

## DISCUSSION

We have identified through TILLING a new stable zebrafish model of SOD1 ALS. Our model, which has normal expression levels of the mutant *sod1* gene and protein, is different to previously published zebrafish *sod1* models, which used either human mutant *SOD1* mRNA injected into single-cell-stage embryos, producing a dose-dependent axonopathy in the developing zebrafish embryos (Lemmens et al., 2007), or transgenic G93R *sod1* zebrafish, which show NMJ alteration and swimming defects (Ramesh et al., 2010) similar to those shown in this study.

Although the recent genetic discoveries in the field of ALS particularly relating to alterations in *TARDBP* (Kabashi et al., 2008), *FUS/TLS* (Kwiatkowski et al., 2009) and *C9ORF72* (DeJesus-Hernandez et al., 2011; Renton et al., 2011) do identify previously unknown disease mechanisms requiring investigation, clearly SOD1 remains of key importance for understanding motor neuron degeneration and there is a need for better models to complement the repertoire of models currently available. Our T70I mutant zebrafish model replicates several of the features of human ALS, expresses the mutant protein at physiological levels and has properties that facilitate high-throughput drug screening.

### T70I mutation located in zinc-binding region of *sod1*

The T70I *sod1* mutation occurs immediately adjacent to the conserved region in the zinc-binding loop of the Sod1 protein (Nordlund et al., 2009). The zinc ion is crucial for Sod1 enzyme stability, and the pathogenic mechanism of the T70I mutation might be due to the alteration of the zinc-binding properties of T70I Sod1. Mutations in the zinc-binding loop and the zinc-binding sites, such as G72C (Stewart et al., 2006), D76Y (Andersen et al., 1997) and H80A (Alexander et al., 2002), are associated with classical ALS phenotypes. Cytoplasmic inclusions positive for both ubiquitin and SOD1 were identified in lower motor neurons and anterior horn cells at post mortem in G72C (Stewart et al., 2006) and H80A (Alexander et al., 2002) ALS patients, providing evidence that mutations affecting the region of the zinc-binding loop of SOD1 cause protein instability, incorrect dimerisation of SOD1 and aggregation of the SOD1 protein.

### T70I Sod1 is stable and has a partial reduction in dismutase activity

Mutation in SOD1 causes ALS by a toxic gain-of-function mechanism (Reaume et al., 1996). A wide range of enzymatic activity of SOD1 has been described, and the different levels are associated with different mutations within the *SOD1* gene. Despite this observation, all SOD1 mutations cause fALS, implying that the normal or impaired enzymatic function of SOD1 is irrelevant to the pathogenesis and progression of ALS. Our data are consistent with these earlier observations. We have demonstrated that T70I Sod1 is stable and that this mutation is associated with a partial reduction in dismutase activity. The native zebrafish Sod1 was observed to have an apparent weight of ~65 kDa, double that of the dimerised human and mouse SOD1. Although an unexpected finding, a tetrameric form of Sod1 has previously been described in teleost skin (Nakano et al., 1995), and our findings suggest the possibility that zebrafish Sod1 also exists in a tetrameric form.

### T70I *sod1* mutation is associated with susceptibility to oxidative stress

In mammalian ALS models, SOD1 mutation confers susceptibility to oxidative stress and this mechanism has been identified as one of the key pathways in ALS pathogenesis (Barber and Shaw, 2010). We have demonstrated that NSC34 cells transfected with zfT70I *sod1* were viable when grown under normal conditions and that, upon serum withdrawal for 6 hours, the zfT70I-*sod1*-transfected cells demonstrated increased oxidative stress as measured by reactive oxygen species (ROS) production in a dichlorofluorescein (DCF) assay. The response to oxidative stress of the zfT70I-*sod1*-transfected NSC34 cell line was comparable to that observed in the established hG93A-*SOD1*-transfected NSC34 cell line (Barber et al., 2009). These data support the pathogenicity of the zfT70I mutation via a toxic gain of function mediated in part through an increased susceptibility to oxidative stress.

### *T70I* zebrafish display an early NMJ phenotype and a late motor phenotype

Since 2004, there has been an increasing body of evidence that points to defects in the NMJ as one of the primary pathological events in ALS (Fischer et al., 2004; Dupuis et al., 2009). The T70I model exhibits early abnormalities at the NMJ. At 11 dpf there is a significant reduction in colocalisation of α-bungarotoxin and SV2 in the interseptal region, between homozygous T70I *sod1* zebrafish and their WT and heterozygous clutch mates. This observation might be due to either a failure of the NMJs to form correctly during embryogenesis, or due to a later disruption of the NMJ, followed by the dying back of the axon. Further investigation will be required to determine which of these mechanisms is operating. Our data are consistent with evidence from other studies that point to NMJ alteration as an early pathogenic event and support the validity of the T70I zebrafish model (Fischer et al., 2004; Dupuis et al., 2009; Dupuis and Loeffler, 2009; Ramesh et al., 2010).

It should be noted, however, that the NMJ of zebrafish are not distinctly organized, unlike the human and mouse NMJ, which form pretzel-shaped structures. The NMJs in zebrafish are present all along the axonal tracts. Thus, characterising denervation in zebrafish is more difficult than in mammalian systems. Additionally, owing to the polyneuronal innervation in zebrafish, individual muscle fibres that are totally devoid of NMJs are rarely present until the terminal stages of disease (Ramesh et al., 2010). Thus, reduced colocalisation and/or reduced pre/post-synaptic (SV2 and α-bungarotoxin) labelling is used as an indicator of NMJ abnormality in larval zebrafish (Boon et al., 2009; Ramesh et al., 2010).

The early NMJ phenotype does not lead to a measurable impairment of motor function until the adult zebrafish reach ~20 months of age. It is possible that the polyneuronal innervation of zebrafish muscle or the regenerative capabilities of zebrafish neurons prevent the onset of motor symptoms at a younger age (Fleisch et al., 2011). At 20 months, a motor defect is clearly observed and the adult homozygous T70I *sod1* zebrafish spend a greater proportion of time at the bottom of the tank and in the ‘resting’ phase of motor activity, compared with their WT clutch mates. This increased time spent in the resting phase might correlate with a progressive loss of motor function that is developing in the homozygous T70I *sod1* zebrafish.

The slow degenerative process in T70I zebrafish coupled with high regenerative capacity of the zebrafish makes it difficult to determine exactly when motor neuron loss starts in these mutants. Hence, adults were chosen for quantification of motor neuron loss because we expected to see the maximum difference between the mutants and WT at this stage. Although we cannot rule out neuronal loss earlier than 2 years, we clearly demonstrate that older zebrafish show the characteristic hallmark features of ALS, i.e. age-related loss of motor neurons, thus validating the model.

ChAT staining is the gold standard measure of cholinergic motor neurons in all animal species. Becker et al. have shown different sizes of motor neurons in the zebrafish spinal cord (Becker et al., 2004). Small motor neurons are generally thought to represent young motor neurons, which become larger as they mature. The total number of motor neurons in the homozygous zebrafish spinal cord was significantly reduced as compared with the WT zebrafish (Fig. 4). To ensure that only large mature motor neurons were counted, a size threshold was set and only motor neurons with an area greater than 200 μm^2^ were counted. As expected, even the large mature motor neurons were reduced in the mutants as compared with the WT spinal cord. Thus, this reduction in ChAT motor neurons truly reflects the death of motor neurons in the mutants.

The loss of motor neurons in the homozygous T70I *sod1* mutant adult zebrafish replicates a key pathological feature of the disease in humans. This, along with the development of a later adult phenotype, resembles human ALS more closely than the aggressive murine models and this might in part be due to the more physiological level at which the mutant protein is expressed.

### T70I zebrafish can be used to identify potential therapeutic targets for ALS

Susceptibility to oxidative stress at 24 hpf was chosen as the preferred readout for drug screening experiments because it represents a clear and easily measurable outcome that could be performed simultaneously in large numbers of zebrafish embryos. The other potential readouts, the NMJ phenotype and motor phenotype, were considered too time consuming to be practical within a future high-throughput screen. We identified two antioxidant compounds, apomorphine-S and olesoxime, to assess the feasibility of utilising the T70I *sod1* embryos in drug screening assays. Both apomorphine-S and olesoxime give rise to significant protection to the WT and homozygous T70I *sod1* zebrafish embryos against oxidative stress. These data indicate that that the susceptibility of T70I *sod1* embryos to oxidative stress can be used as a readout in a future large-scale drug screening programme.

Through TILLING we have developed a novel zebrafish model of ALS caused by to T70I *sod1* mutation. The model demonstrates key features of ALS: an early NMJ phenotype, susceptibility to oxidative stress and an adult-onset motor phenotype. We have demonstrated that the susceptibility of T70I *sod1* embryos to oxidative stress can be used in a drug screening assay to identify compounds that merit further investigation as potential therapies for ALS.

## MATERIALS AND METHODS

### Materials

All general chemicals and reagents, unless otherwise stated, were obtained from Sigma-Aldrich (Poole, Dorset, UK). Polymerase chain reaction (PCR) primers were purchased from Sigma-Genosys (Haverhill, UK) or Integrated DNA Technologies (IDT; Coralville, USA). Tissue culture consumables were from Gibco Life Technologies (Paisley, Scotland) and tissue culture plastics were from Greiner (Gloucestershire, UK).

### Zebrafish husbandry

Adult and larval zebrafish stocks were kept at 28.5°C in the University of Sheffield zebrafish facility and bred according to established protocols (Westerfield, 2000). All zebrafish husbandry and experimental procedures were performed in accordance with the UK Animals (Scientific Procedures) Act 1986.

### TILLING

Male Tübingen Longtail (TL) zebrafish were treated with a 6-week course of 3.0 nM ENU in order to mutagenise their gametes. These fish were subsequently bred with female TL zebrafish, and the resulting F_1_ progeny were allowed to reach adulthood. This F1 generation formed the TILLING library (Wienholds et al., 2002).

Nested primers were designed for amplicons of interest using the Laboratory Information Management System for Identification of Mutations by Sequencing and TILLING (LIMSTILL) website (http://limstill.niob.knaw.nl/), with the internal set of primers being M13-tagged for ease of sequencing by the Sanger Institute.

### Cell culture

Neuroblastoma × Spinal Cord hybrid 34 (NSC34) cells (Cashman et al., 1992) were cultured using standard techniques (Cookson et al., 1998). ZfWT and zfT70I *sod1* inserts were cloned into the pIRESneo vector and used to transfect NSC34 cells. The selection pressure G418 was used throughout to ensure selection at a concentration of 250 μg/ml.

### Cellular oxidative stress assays

NSC34 cells were grown in 96-well tissue culture plates in phenol-red-free DMEM containing 10% FBS until 30–40% confluent. Oxidative stress was induced by 3-hour serum withdrawal. Cytosolic ROS levels were measured using DCF fluorescence (Barber et al., 2009). Carboxy-H_2_DCFDA (Molecular Probes, Paisley, UK) was added to NSC34 cells to a final concentration of 5 μM, and DCF fluorescence was read at Ex485 nm/Em530 nm after 1 hour. Cell death was simultaneously measured by adding ethidium homodimer-1 (EthD1, 0.3 μM, Molecular Probes) to the culture medium, and the fluorescence was measured at Ex530 nm/Em645 nm. When different cell lines were compared, the DCF results were normalised to the cell number, which was determined by measuring EthD1 fluorescence after the cells had been freeze-thawed.

### Western blotting

Western blotting was carried out according to standard protocols. Owing to co-migration of zfSod1 and native mSOD1 in the NSC34 cells, densitometry was required to determine the level of overexpression. The film blots were placed inside an Alphainnotech MultiImage Light Cabinet (Flowgen, Berks, UK), photographed and densitometry carried out using Alphainnotech software (AlphaImager 1220 v5.1). Ratios of protein of interest to an actin loading control were calculated and, from these ratios, the level of overexpression of Sod1 could be determined.

### Native gel for enzymatic activity

Samples were placed into a non-reducing lysis buffer [1× PBS, 1 Proteinase Inhibitor Cocktail (PIC) tablet (Roche, Basal, Switzerland) per litre] and sonicated [Soniprep 150, MSE (UK) Ltd, London] for 30 seconds. Prior to running the samples on the polyacrylamide gel, the samples were added to a 2× loading buffer (160 mM Tris-HCl pH 7.0, 20% glycerol, 0.0025% Bromophenol blue) and incubated at room temperature (RT) for 10 minutes.

Protein samples were run on 8% acrylamide gels. Molecular weight standards were added to determine protein size, along with the 25 μg protein samples. An identical gel was run to Coomassie stain to ensure equal loading was achieved.

The gel was first soaked in 1.23 mM NBT for 15 minutes at RT on a shaker, briefly washed in H_2_O, before being soaked again in the dark in 100 mM potassium phosphate buffer (pH 7.0) containing 28 mM TEMED and 28 μM riboflavin for another 15 minutes at RT on a shaker. The gel was briefly washed again in H_2_O, before being placed onto a transilluminator to develop (Beauchamp and Fridovich, 1971).

### Embryonic survival assay

Dechorionated 24 hpf T70I homozygous and WT embryos were placed into Petri dishes and H_2_O_2_ was added to a final concentration of 5 mM to half of the Petri dishes and incubated at 28°C for 24 hours before counting for survival as judged by the presence of a heartbeat at 48 hpf.

### Drug treatment

Apomorphine-S was identified through an in-house screening cascade of CNS-penetrant Nrf2 activators (Barber et al., 2009), and has been observed to delay the onset of ALS symptoms in transgenic G93A *SOD1* mice (Mead et al., 2013). Olesoxime has been demonstrated to prolong motor neuron survival via modulation of the mitochondrial permeability transition pore and was recently evaluated in a phase 3 trial in human ALS (Bordet et al., 2007). Dose-response curves were conducted to establish an optimal drug concentration. These were done by assessing the toxicity of the drug on three plates of 60 embryos at various doses and represented as percent survival. Optimal doses were then added at gastrulation (final concentration of 1% DMSO), and exposure continued for the duration of the assay. 5 mM H_2_O_2_ was added as the oxidative stressor at 24 hpf and survival was measured at 48 hpf.

### NMJ staining

NMJ staining was carried out using the protocol described by Ramesh et al. (Ramesh et al., 2010). WT and T70I homozygous embryos were stained in the same tube to accurately compare the NMJ staining. Heads were removed prior to mounting for genotyping. The interseptal region is defined as the region in between the myoseptal regions. This is the region where the longer axons terminate (Myers et al., 1986). The ventral half of the interseptal region was used for analysis.

### ChAT immunohistochemistry

ChAT immunohistochemistry was performed as described (Clemente et al., 2004). Briefly, fresh frozen sections (20 μm) of spinal cord were collected from 3-year-old zebrafish that had been fixed in 4% PFA in 0.1 M phosphate buffer (PB) (pH 7.4) for 10 minutes. The sections were washed twice in 0.1 M PB (pH 7.4) for 5 minutes per wash. To decrease background staining, the sections were incubated in 2% H_2_O_2_/PBS for 20 minutes. Sections were washed three times in PBDT with 0.2% Tween 20 for 5 minutes, incubated in PBDT with 0.2% Tween and 10% normal donkey serum (NDS) for 2 hours at RT, followed by incubation in goat polyclonal anti-ChAT antibody (1:125 in PBDT with 0.2% Tween and 10% NDS, Chemicon International) for 2 days at 4°C. Sections were washed four times with PBST with 0.2% Tween 20 for 15 minutes each and incubated in biotinylated donkey anti-goat secondary antibody (1:250 in PBDT with 5% NDS, Santa Cruz Biotechnology) for 2 hours at RT. Sections were washed four times with PBST with 0.2% Tween 20 for 15 minutes each and washed twice with 0.01 M PB (pH 7.4) for 15 minutes. Sections were incubated in Vectastain RTU reagent (Vector Labs) for 90 minutes and were washed four times with 0.01 M PB (pH 7.4) for 15 minutes each. The reaction product was visualized with 0.025% 3,3-diaminobenzidine (DAB) and 0.0033% H_2_O_2_ in 0.01 M PB (pH 7.4) or 0.2 M Tris (pH 7.6). The course of the reaction was monitored and stopped by washing in PBST. Sections were serially dehydrated for 10 minutes each in 70% and 100% EtOH, cleared in xylene for 15 minutes, and mounted with Permount (Fisher Scientific). Sections from the dorsal fin region were blinded to genotype and each section was counted for ChAT-positive motor neurons with an area greater than 200 μm^2^. A minimum of 40 serial sections were counted from three fish for each genotype (Ramesh et al., 2010).

### Adult movement analysis

Locomotor activity was assessed using the ZebraLab system (ViewPoint, Lyon, France) (Tierney, 2011). T70I homozygous mutants and WT animals were imaged ten at a time in a tank and filmed simultaneously. An opaque screen separated the two tanks so that the fish from the two tanks did not influence each other’s behaviour. The fish were allowed to acclimatize in the tank for 15 minutes and then the activity was recorded continuously for 15 minutes. The same animals were analysed again after 7 days and three independent observations from these fish were recorded. The system consists of a digital camera connected to a computer with the ZebraLab software (ViewPoint, Lyon, France), which was able to process the global activity and the average centre of mass of a group of fish in a tank. To improve detection, a ZebraSquare illumination device (ViewPoint, Lyon, France) was used. This consists of an infrared lighting unit that increases contrast between the fish in the tank and the background, especially in totally dark conditions. The images were digitized with an 800×600 pixels definition. Each pixel is coded on 256 grey levels. The images were processed in real time at 25 frames per second. Global activity was classified into three different categories based on user-defined thresholds. The classification is made between inactivity, normal activity and hyperactivity. The centre of mass of the group of fish in relation to the depth of the tank was assigned into equal horizontal thirds (top, middle and bottom).

## Supplementary Material

Supplementary Material
