# Supplementary material for: A new zebrafish model produced by TILLING of SOD1-related amyotrophic lateral sclerosis replicates key features of the disease and represents a tool for in vivo therapeutic screening
Source: Dis Model Mech. 2013 Oct 2;7(1):73–81. doi: 10.1242/dmm.012013 (PMC3882050; doi:10.1242/dmm.012013)
Supplement: Supplementary Material [file supp_7_1_73__index.html]

A new zebrafish model produced by TILLING of SOD1-related amyotrophic lateral sclerosis replicates key features of the disease and represents a tool for in vivo therapeutic screening — A new zebrafish model produced by TILLING of SOD1-related amyotrophic lateral sclerosis replicates key features of the disease and represents a tool for in vivo therapeutic screening — Supplementary Material 

# A new zebrafish model produced by TILLING of SOD1-related amyotrophic lateral sclerosis replicates key features of the disease and represents a tool for *in vivo* therapeutic screening

## DMM012013 Supplementary Material

**Files in this Data Supplement:**

- **Supplementary Material PDF**
