# Supplementary material for: A new zebrafish model produced by TILLING of SOD1-related amyotrophic lateral sclerosis replicates key features of the disease and represents a tool for in vivo therapeutic screening
Source: Dis Model Mech. 2013 Oct 2;7(1):73–81. doi: 10.1242/dmm.012013 (PMC3882050; doi:10.1242/dmm.012013)
Supplement: Supplementary Material [file supp_012013_DMM012013.pdf]

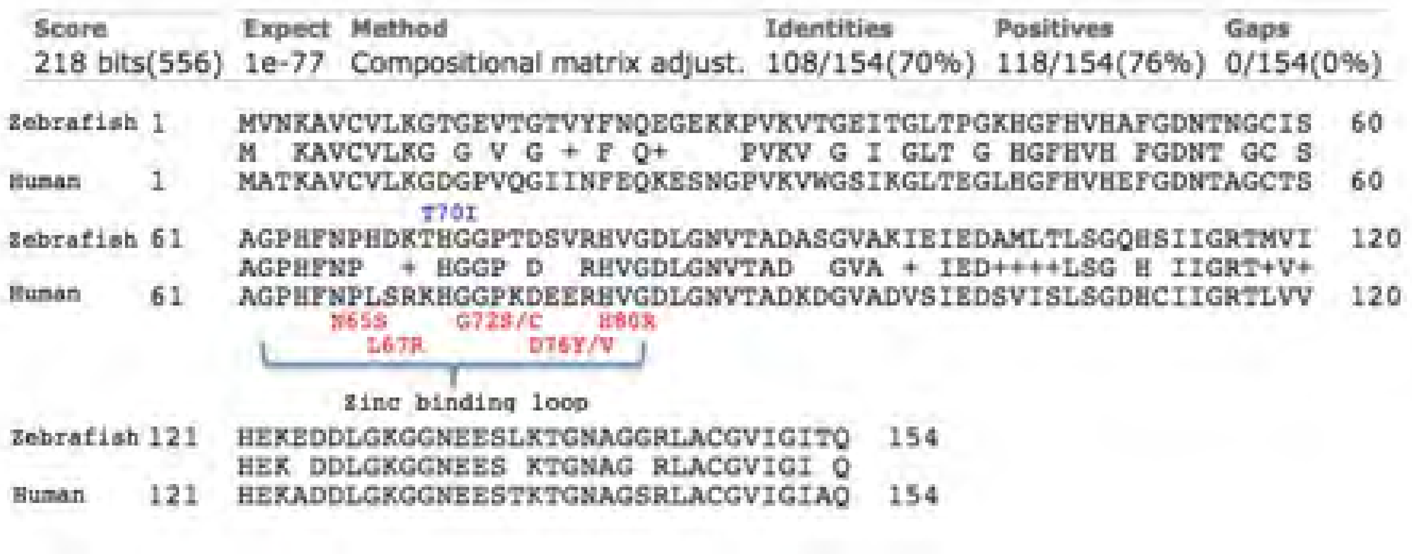

Supplementary Fig. 1. A Blast P of zebrafish and human SOD1, annotated to show the location of the T70I mutation in zebrafish Sod1 (in blue), and also the location of disease causing mutations in the zinc binding loop of human SOD1 (in red).
